# The War on the Hudson

**Published:** 1883-06

**Authors:** 


### HOMŒOPATHIC PHYSICIAN,

A MONTHLY JOURNAL OF MEDICAL SCIENCE.

If our school ever gives up the strict inductive method of Hahnemann, we are lost, and deserve only to be mentioned as a caricature, in the history of medicine."—CONSTANTINE HERING.

Vol. III. JUNE, 1883. No. 6.

### EDITORIAL.

THE WAR ON THE HUDSON.—The war in New York over the "New Code" is being waged with relentless severity. The "New Coders" have captured (for the present) the State Society and the New York County Society. The "Old Coders" have, by a tricky stratagem of their leader, Austin Flint, Jr., captured the New York Academy of Medicine. Each side has formed an association and issued addresses for the promotion of its views. Great bitterness has been aroused between the contending brethren. Dr. Barker proclaims Dr. Flint's tactics a "disgraceful, abominable trick, and only fit to be undertaken by a low ward politician." Pretty severe language for a cultivated, scientific regular! The "New Coders" are headed by Drs. Barker, Jacobi, Agnew, Roosa, etc., the "Old Coders" by Drs. Flint, Gouley, Purdie, etc. The New York press seems to be decidedly with the "New Coders." As an incident of the *war*, we may mention that the Bellevue College got in the first lick and by it decapitated Prof. Howe, said Professor being a staunch "New Coder," while Bellevue is rigidly regular and orthodox. The Pennsylvania State Society at its recent meeting indorsed the Old Code, and proclaimed the "New Coders" to be "in rebellion against constituted authorities!" The next campaign will be fought in Cleveland at the meeting of the American Medical Association. This Association refused last year to admit delegates from the New York *State* Society, but admitted those from the *County* Societies. A sharp way of getting around a difficulty.

The homœopathists can afford to serenely view the war over a nonsensical code, little caring whether Dick or the Devil wins. To sensibly minded persons it will seem odd that educated gentlemen should need a "code" at all.

---

THE RENEGADE PRAISES THE MONGRELS:—John C. Peters extends the right hand of fellowship to the American Institute. In the *New York Herald* we find the following eulogy of mongrelism: "Some of the New Code men recognize," said Dr. Peters, "that the homœopaths of to-day are in a very different position from what they formerly were. They have rejected infinitesimal doses and the disgusting theory about psora. They use many of the remedies and *doses* of the regular school; are no longer mere sectarians, but more or less educated and honorable physicians, who have preferences for some well-known, powerful, and efficient remedies. The same change of opinions holds good with regard to some of the eclectics. These were formerly botanic physicians, who would use no mineral remedies, and believed that a good medical and surgical education was not necessary to form an accomplished and successful physician. Some of the eclectics are now more or less educated and reasonable men. With these better classes of homœopaths and eclectics the New Code men are willing to affiliate, meet in council, and even admit into the regular medical societies. They wish to treat this matter precisely as they did the female physician question. When they found that they could not prevent many women from studying and practicing medicine, they took the best by the hand, met them in consultation, received them into the best medical societies, developed and regulated a movement which they could not wholly stop, and thus brought order out of chaos.

ALL IN THE USE OF MEDICINES.

"The New Code men simply recognize the fact that every remedy is good for something, if one only knows how to use it. That it is not only the right but the duty of every physician to use those remedies which he finds most useful. That as soon as they cease to band themselves closely or exclusively into schools, systems, or sects, the *homœopaths and eclectics are more or less regular physicians in a somewhat false and uncomfortable position,* from which they should be aided and helped. Some of the New Code men even think that the regular profession is merely the biggest, most powerful, and perhaps the most arbitrary of all the sects in medicine, while it should be catholic and generous enough to adopt everything which is good in all systems, and countenance and conciliate all of their adversaries who are well educated and honorable.

"Above all," said Dr. Peters, "the New Code men are struggling for freedom of thought and action—for liberty in scientific research. They are not afraid from all they will learn from all systems that they will cure too many people or relieve too much suffering. They are firm in their reliance upon the principles and practice of the regular school, but admit that experience is entitled to weight and consideration, as well as reason and theory. They want unity in the whole medical profession, and can only be driven into rebellion when governed by narrow-minded, arbitrary, and interested officers."

After such a cordial invitation, the American Institute will surely do its best to come out of its "somewhat false and uncomfortable position." The Institute has of late years given evidence of a yearning for "liberality" in thought and action. Their pleadings have, at last, been heard, their repentance has been accepted, and now the renegade, John C. Peters, extends them a pitying pardon —pardoned because they, the better class of homœopaths, have "rejected infinitesimal doses and the disgusting theory about psora." Now, to prove that they can appreciate the benevolence and kindness of the allopaths, let the "liberals" pass the following resolution (of Dr. Minor's), then drop their "distinctive name" and be *absorbed*. Let the Institute adopt the following, and all may yet be happy:

"*Believing that the formula* SIMILIA SIMILIBUS CURANTUR *forms the best general guide in the selection of remedies, we do not recognize it as a law, nor follow it as an exclusive method, but exercise the right belonging to every educated physician to make practical use of any established principle in medical science, and to employ any facts in therapeutics that are founded on experiments and confirmed by experience, so far as in our judgment they may tend to promote the welfare of those under our professional care.*"

Dr. Minor was honorable enough to resign from homœopathic societies, with which he could no longer conscientiously affiliate; let the Institute imitate Dr. Minor and drop its distinctive title, "Homœopathy," from its name.

Hume tells us how Cromwell, becoming disgusted with "Barebone's Parliament," sent an officer to disperse this, the tail end of that once powerful Parliament. The officer "asked them what they did there. 'We are seeking the Lord,' said they. 'Then you may go elsewhere,' replied the blunt soldier, 'for to my certain knowledge, He has not been here these many years!'" So it is with the once great American Institute—it has degenerated, and Homœpathy has not been represented there for these many years.

